# Effects of CNC Machining on Surface Roughness in Fused Deposition Modelling (FDM) Products

**DOI:** 10.3390/ma13112608

**Published:** 2020-06-08

**Authors:** Mohammadreza Lalegani Dezaki, Mohd Khairol Anuar Mohd Ariffin, Mohd Idris Shah Ismail

**Affiliations:** Department of Mechanical and Manufacturing Engineering, Universiti Putra Malaysia, Selangor 43400, Malaysia; gs51329@student.upm.edu.my (M.L.D.); ms_idris@upm.edu.my (M.I.S.I.)

**Keywords:** additive manufacturing, fused deposition modelling, computer numerical control, surface roughness, machining, FDM, CNC, Polylactic acid (PLA)

## Abstract

Fused deposition modelling (FDM) opens new ways across the industries and helps to produce complex products, yielding a prototype or finished product. However, it should be noted that the final products need high surface quality due to their better mechanical properties. The main purpose of this research was to determine the influence of computer numerical control (CNC) machining on the surface quality and identify the average surface roughness (*Ra*) and average peak to valley height (*Rz*) when the specimens were printed and machined in various build orientations. In this study, the study samples were printed and machined to investigate the effects of machining on FDM products and generate a surface comparison between the two processes. In particular, the block and complex specimens were printed in different build orientations, whereby other parameters were kept constant to understand the effects of orientation on surface smoothness. As a result, wide-ranging values of *Ra* and *Rz* were found in both processes for each profile due to their different features. The *Ra* values for the block samples, printed samples, and machined samples were 21, 91, and 52, respectively, whereas the *Rz* values were identical to *Ra* values in all samples. These results indicated that the horizontal surface roughness yielded the best quality compared to the perpendicular and vertical specimens. Moreover, machining was found to show a great influence on thermoplastics in which the surfaces became smooth in the machined samples. In brief, this research showed that build orientation had a great effect on the surface texture for both processes.

## 1. Introduction

Nowadays, manufactured products are getting more complex and requiring more tasks undertaken in their product development stage. In particular, curves and specific designs exist in various applications, such as aircraft industries, construction, and automotive companies [1,2]. For example, vehicle components are improving and many difficulties have thus been noted in producing complex products in the industry. The features of these components are mostly complex, consisting of fillets, chamfer, and circles. Therefore, complex products are vital in manufacturing industries. Rapid prototyping (RP), which is formerly known as additive manufacturing (AM), is the foundation for a large number of manufacturing techniques involving fusing of materials layer-by-layer. By utilising AM in various applications, such as aerospace, construction [3], medical [4], and automation [5], it allows the engineers to enhance the end-user and assembly products accordingly [6]. Cabin accessories in airplanes, scale models in the construction field, implants and prosthetic hand in the medical field, rear wing, jigs, and fixtures in automotive and manufacturing fields, and toys in the assembly section are a few examples which can be handled by AM. Furthermore, reducing the material waste, time, cost, and errors are some of the main advantages of AM, whereby it is possible to fabricate complex parts using such technologies [7]. Calignano et al. [7] reviewed and analysed different types of AM processes to focus on process capabilities and their features to print various products. Thus, this research identified the advantages and disadvantages of AM processes. Its processes are divided into six categories, namely: directed energy deposition [8], material extrusion [9], material jetting [10], powder bed fusion [7], sheet lamination [11], and vat photopolymerisation [12]. In the process, exclusive software programs are employed to monitor and obtain the optimum parameters towards improving product quality by changing parameters such as layer thickness, temperature, and build orientation [13]. Ariffin et al. [13] analysed accuracy and surface texture of FDM samples by comparing CuraEngine^®^ and Slic3er^®^ softwares. They found out CuraEngine^®^ was more accurate while Slic3er^®^ was better for hanging structures. Moreover, a wide range of base materials such as metals, plastics, composites, ceramics, and more recently, bio-materials can be used in AM [7,14,15]. These materials can be powder, liquid, and solid in such processes, whereas plastics are deemed as superior compared to metals as a base material for the performance and ease of manufacturing [16]. Moreover, the most commonly used material in 3D printers is polymer due to its diversity and ease of adoption [14]. In general, fused deposition modelling (FDM) is one of the most typically utilised AM techniques, which is similar to a hot glue gun and offers low cost and ease of use without any chemical and harmful post-processing requirements [17,18]. FDM (and AM in general) gives more freedom in terms of design compared to conventional subtractive manufacturing techniques [19]. For example, Panda et al. [19] evaluated the FDM process and its parameters to find out the strengths and limitations such as warping in printing various samples. Furthermore, it should be noted that FDM is a capable process for building composite based materials [20,21,22], geopolymers [23], metal alloys [24,25], and biocomposites [26]. This method consists of processing a Stereolithography (.STL) file, which will then mathematically slice the designed model to initiate the process. Then, molten material is extruded and deposited on a flat surface, namely either the printer tray or another layer of printed material. As soon as the material leaves the nozzle where it is extruded, it is solidified and bonded to the previous printed layer, resulting in a fully-built piece using thermoplastic bottom-up [27]. However, severe temperature differences will lead to defects such as warpage and shrinkage in the process [28,29]. Kuo et al. [28] developed a closed chamber to control the temperature and analysed FDM parameters’ effects on samples. By this technique, they have found optimal process parameters to eliminate warpage. Moreover, support material is necessary for AM, especially when a particular geometry has no material underneath with the print platform. Regardless, support material waste is a significant issue in this process due to printing parameters, whereby following its completion, the supports should be removed by post-processing techniques [30]. In the past few years, developments have been made in the FDM machine, which can build a product with high accuracy and less error [31,32,33]. For example, developing FDM 3D printers to 4-axis and 5-axis or changing the cartesian coordinate robot to a delta robot would give more freedom and tight tolerances [31,33]. These changes can reduce dimensional errors, defects, and printing time. However, in some cases, AM is not the best solution to produce products, thereby rendering them to be easily milled or turned without cracks by using computer numerical control (CNC) machines in a proper condition [18,34]. Many parameters can affect the final shape in FDM compared to other AM processes, such as layer thickness, build orientation, raster angle, infill pattern, infill density, nozzle temperature, nozzle angle, etc. Each of these elements poses a great effect on the final surface smoothness. Moreover, high surface quality is a vital factor, resulting in aerospace, automotive, and manufacturing companies attempting to reach a high standard of quality. Manufacturing industries, in particular, pursue a particular aim to increase the product quality and reduce the development time in manufacturing processes. Following the improvement of design tools such as computer-aided design (CAD) over the past few decades, many obstacles have been identified in the manufacturing process in order to make a specific product. For example, computers allow complex components to be drawn but the parts cannot be easily built using conventional methods. Therefore, new processes such as AM bring many advantages at a lesser cost [8,35]. Regardless, mechanical properties, surface quality, and product structure are some of the main concern in machining and AM processes, resulting in many studies geared towards improving machine’s capabilities [36,37,38]. Despite AM being a potential technique, many challenges exist during the process [5]. By selecting the optimal process parameters in both processes, high precision and accuracy can thus be achieved [39,40]. Salmi et al. [40] examined the effect of build orientation in different AM processes to find out their capability and performance.

Producing parts with tight tolerance and high accuracy are the main features of CNC machines, whereby developing efficient algorithms makes it possible to eliminate the faults within a precision engineering process [41,42]. Modelling and simulation techniques are thus used to reduce the costs and time required to design CNC machines, thereby yielding many advantages financially. Furthermore, CNCs have a strong tendency to form aggregate structures, making it difficult for them to disperse homogenously in polymer matrices [43]. Therefore, specific feed rate, cutting speed, cutting tool, and depth of cut are the requirements of machining due to material characteristics in order to attain their functional features [44,45]. Meanwhile, product roughness can be predicted by surface measurement with specific equipment [46,47]. In the manufacturing process, controlling the surface roughness is difficult, especially for FDM parts [48]. By decreasing the surface roughness, the product cost will increase. Based on ISO 4287:1997, some parameters such as roughness average (*Ra*), average maximum height of the profile (*Rz*), skewness (*Rsk*), kurtosis (*Rku*), and more are presented to understand surface roughness clearly [46]. The problem arises when surface quality is compromised; it is a must for some products to meet the specified quality of surface finish.

Thermoplastic machining has been used to reach better accuracy and quality. To eliminate defects in 3D printed samples and meet smoothness in surface texture. CNC machining was used in this study due to tight tolerances. Thus, a CNC machine can meet lower tolerance and high quality in products. Also, staircase defect is the main issue in AM due to layer by layer binding process. This defect is changing in different build orientations due to angle that the machine starts to print. Further, build orientation is an important parameter in FDM and it affects surface quality and mechanical properties. Thus, this study investigated the build orientation and two processes that can be combined and used in hybrid manufacturing (HM). One the other hand, machining of composite polymers and thermoplastics requires specific features, parameters, and tools due to their low melting point [44,49]. Some difficulties may thus be faced while completing the objectives. In contrast, polylactic acid (PLA) does not have the same mechanical properties in all directions (i.e. not isotropic) [11]. Moreover, plastics commonly melt during the machining process, and machining complex plastic products are difficult due to the CNC machine’s limitations towards obtaining complex shapes [2]. Additionally, the products surface quality in the FDM process is not as good as other AM processes, such as Stereolithography (SLA) [50]. 

Unfortunately, not much research has been done in the topics of smooth machining of FDM products and PLA material. Therefore, this study investigates the machinability of FDM products based on various build orientations to distinguish the surface quality after and before machining. In particular, surface roughness testing is conducted on specimens manufactured in both processes and the generated data are used to investigate the relationship between the process parameters and surface quality. Furthermore, it investigates the optimum build orientation to attain better surface quality in both processes by analysing the average roughness (*Ra*) and average peak to valley height (*Rz*). Finally, a comparison between all 3D printed and machined parts is done based on their respective surface roughness behaviour.

## 2. Literature Review 

Surface roughness is a technique that can be used to measure the smoothness of products. It indicates the state of a machined surface. Cover of products, panels, and a vehicle’s body have different appearances which are smooth or rough. The most important 2D parameters are explained here point by point, as shown in Table 1. Finding surface roughness characteristics can be done by using these parameters. Moreover, there are factors that may affect appearance, texture, and mechanical properties, such as friction, fatigue behavior, light reflection, etc. In this study, *Ra* and *Rz* were used to analyse the surface roughness of each sample. 

It can be noted that FDM has the worst surface quality compared to the stereolithography (SLA), selective laser sintering (SLS), and Material jetting (MJ) processes due to the various process parameters that affect surface quality [53,54]. Regardless, improving the product surface can help to increase its stiffness and mechanical properties in which pre- and post-processing techniques can increase or decrease the roughness in FDM based on different conditions. Using various techniques in post-processing will lead to better accuracy and higher precision; however, they are also time-consuming [55]. Chen et al. [56] investigated the manner in which product surface reflectivity can be improved by using a corner cube retroreflector (CCR) array. The work proposed a curved layer fused deposition modelling (CLFDM) algorithm which allowed printing various depth curved thicknesses within a layer. Due to FDM limitation to print complex curves, this technique will be suitable to improve the surface smoothness in complex curves as printing curves requires more support structure, which will affect final accuracy. Furthermore, developing a multi-axis machine and CLFDM algorithm allows complex products to be printed without stair-case defects.

**Figure 1:**
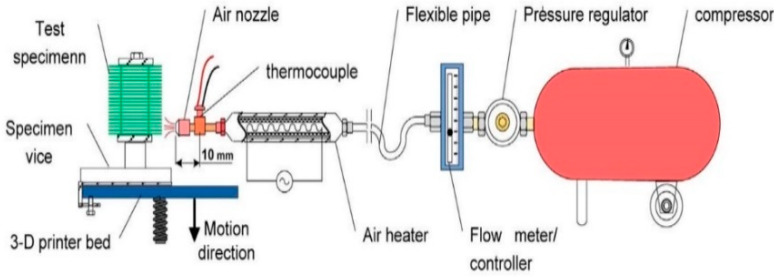
Hot air jet process [55].

Furthermore, Taufik et al. [50] used a carbon dioxide (CO_2_) laser engraving technique to enhance the surface roughness, and the *Ra*, *Rsk*, and *Rku* were studied to assess their spatial features. The results showed that build orientation was increased as the surface roughness parameters were increased. Moreover, the build orientation and laser power yielded an extraordinary interaction based on their *Rsk* and *Rku*, whereas the surface roughness was improved by low *Ra*, as well as negative *Rsk*, and *Rku* > 3. Meanwhile, Adel et al. [57] have developed a hot air jet system to evaluate surface roughness as seen in Figure 1. The results showed that by increasing the jet velocity, the *Ra* decreased significantly up to 88% from a single pass. Similarly, the optimal condition was the highest jet temperature, which was higher than 235 °C and yielded low-to-moderate jet velocity (20 m/s).

Build orientation is a vital element that can affect the dimensional accuracy, build time, and surface finish [58,59]. Meanwhile, Boschetto et al. [60] developed a novel formulation to predict the dimensional deviations of FDM samples, which is thus shown to depend on the deposition angle. For the 90° deposition angle, the dimensional deviation was 0; however, it increased in the range of 0° to 50° and 135° to 180°. Additionally, warping and other defects occurred when the surfaces had small dimensional deviations.

In contrast, another approach has been proposed by Ahn et al. [61] to investigate the surface roughness of FDM products, whereby it is assumed that the filament profile acts as an elliptical curve. A theoretical model based on the surface angle was derived by considering vital factors affecting surface smoothness, which was verified by printing acrylonitrile butadiene styrene (ABS) and Maxum samples and measuring surface roughness in all conditions from various angles. Moreover, Durgun et al. [62] have found that perpendicularly-orientated specimens have the worst surface roughness compared to horizontal and vertical parts.

Moreover, Zhang et al. [63] investigated the effects of layer thickness and road width by using finite element analysis (FEA) in the FDM process, revealing that the level of stress is increased by the increasing road width at smaller layer thickness. Subsequently, in the simulation process and at a constant layer thickness and road width, the distortions increase dramatically. It can be seen that various factors, except FDM parameters, affect the surface roughness. For example, Alsoufi et al. [29] measured the inner and outer surfaces of hollow (0° infill) printed cubes in 0°, 45°, and 90° orientations to find that the 0° specimens have the best surface roughness compared to 45° and 90°. Further, the results show that the outer surfaces are smoother compared to the inner surfaces due to the solidification process. 

Furthermore, one of the subtractive methods in manufacturing is high-speed machining [64], which consists of high feed rate, high spindle turning, and cooling system. Assuming that the 3D printer cannot meet the dimensional requirements, it will be possible to make the parts by machining or other post-processing techniques [65,66]. Thermoplastics machining produces a constant stress factor and tight tolerance compared to injection moulding. Meanwhile, heat is an important factor in plastic machining as increasing the heat will change the cutter tool to a melting tool. By considering the proper cutting speed, feed rate, suitable coolant, and tool design, product melting can be eliminated. Pandey et al. [67] thus developed a statistical model to prognosticate roughness in order to improve the surface finish of products by using hot cutter machining (HCM) by using turning, milling, and grinding processes. As a result, a high correlation (99%) was seen between the model and HCM, whereas a limited cutting tool accessibility during the machining was considered the main limitation of the machining process. Accordingly, other studies have been conducted, showing that the surface quality of FDM parts depends on the deposition angle, cutting depth, and cutting tool [68,69]. Additionally, another study has obtained the values of 0.599 µm average roughness via the parameters of 4138 rpm speed, 1241 mm/min feed rate, and 0.5 mm depth in the machining of polypropylene (PP) products [70]. Meanwhile, to analyse more factors in the CNC machining of polymers, Dhokia et al. [71] have proposed a neural networks (NNs) to predict the surface roughness. The results showed that, at maximum speed, feed rate, and depth of cut, the average roughness was the highest, whereas the intermediate range of CNC parameters would lead to better surface roughness. Moreover, Raju et al. [72] found that by increasing the feed rate, surface roughness was increased and by increasing the spindle speed, roughness was decreased.

Next, Pămărac et al. [73] investigated the optimum CNC milling parameters for machining FDM products, whereby ABS and PLA were the main materials used in the study. Following this, the optimum feed rate for contour and face milling in the end-mill process was found via 3500 rev/min spindle speed. Meanwhile, Prakasvudhisarn et al. [74] developed a model to minimise the machining time and reach the desired surface roughness. Here, support vector machines (SVMs) and particle swarm optimisation (PSO) are used to find the optimum condition. By considering the spindle speed, feed rate, and depth of cut, SVMs are able to predict the surface roughness, whereas the PSO algorithm can successfully model and determine the optimal results. Then, Taufik et al. [75] developed a 3-axis CNC-assisted selective melting tool (SMT) and found that the surface quality of post-treated surface is affected by feed rate. Subsequently, products with lower orientation angles showed poor surface quality compared to a higher orientation angle, which needed a low feed rate. Finally, the *Ra* was found to be 2.173 µm for the SM profile. Also, Krolczyk et al. [76] investigated FDM and turning process to find out surface roughness parameters such as *Ra, Rz, Rp,* etc. They used auto correlation and gradient distribution methods and observed smooth gradient after turning process while the values were fluctuated in a large range for FDM samples.

In view of these research works, using the proper tools such as solid carbide or stainless-steel cutters and coolants like pressurized air and flood (e.g., Astro-Mist 2001A and Trim 9106CS), machining plastic is achievable and feed rate, spindle speed, and depth of cut parameters are found to be dominant in the CNC milling process. In particular, the feed rate, spindle speed, and depth of cut are the main elements affecting the surface roughness during the process. By controlling these factors, it will lead to the desired roughness and product of high quality. Moreover, many post-processing and second processing techniques have been designed to enhance the surface quality. Unfortunately, not much literature is available on the machining of PLA printed products to identify the optimum printed orientation in the machining process.

## 3. Materials and Methods

**Figure 2:**
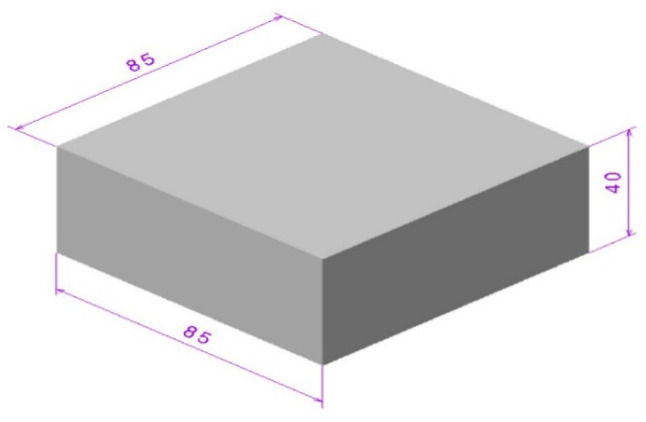
Block sample.

**Figure 3:**
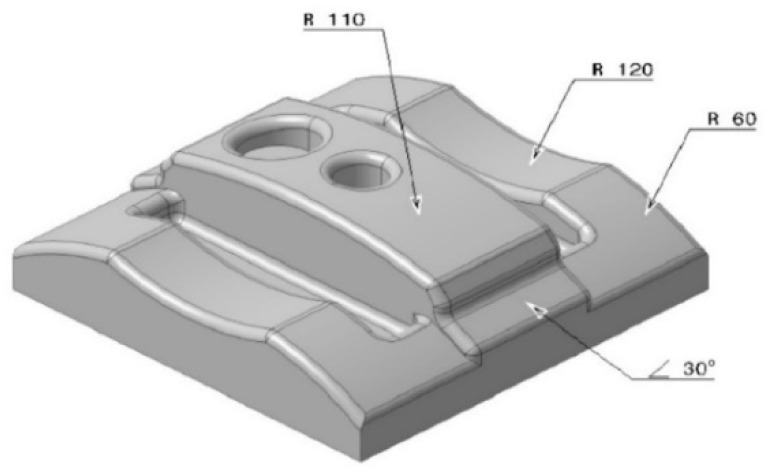
Complex sample’s design.

The study was divided into two parts, whereby the first part consisted of printing the specimens by FDM machine (Ultimaker 2+, Ultimaker B.V, Utrecht, Netherlands), followed by machining the printed bulks by CNC to investigate the effect of various build orientations on surface roughness. Many parameters can affect mechanical properties and final surface quality of the product [77] but this study focused on the build orientation due to its importance in rapid prototyping [78,79]. Bulk rectangle specimens and complex parts were designed in CATIA V5^®^ software (R21, Dassault Systèmes, Vélizy-Villacoublay, France), whereby the dimensions of the artefact were 85 mm length, 85 mm width, and 40 mm height, as shown in Figure 2. This part was printed by the FDM machine for further activities. The cube was then chosen to determine the surface roughness and identify the quality of final products in various orientations before and after machining. The specific features such as fillets, holes, curves, and pockets are shown in Figure 3. These features were used to investigate the effect of FDM and CNC processes on the small elements in thermoplastics. Additionally, curve surfaces were used due to their application in various industries in order to identify the manner in which machining would affect the curves.

**Figure 4:**
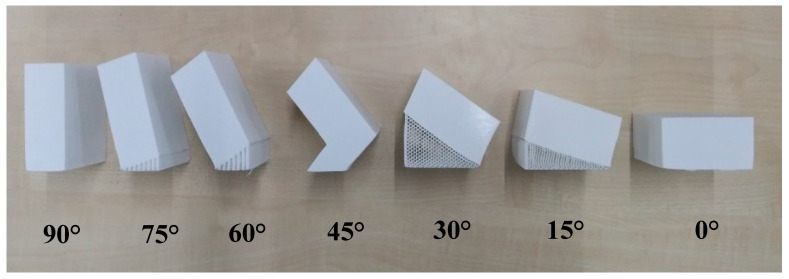
Blocks in different build orientations.

Meanwhile, the Ultimaker 2+ machine (Ultimaker B.V, Utrecht, Netherlands) was used to print fabricates in various build orientations. First, the sample was sliced by Cura^®^ software (Cura 4.4, Ultimaker B.V, Utrecht, Netherlands) using default setting parameters for the printing process, whereas White PolyLite^TM^ (Polymaker, Shanghai, China) Polylactic acid (PLA) was used to print the products. The goal was to identify the optimum build orientation before and after machining of the FDM parts; therefore, other elements were kept constant during the process. Figure 4 shows the products are built in seven orientations ranging from 0° to 90°. The values of 0.4 mm for extruder diameter, 2.85 mm filament diameter, 0.15 mm layer thickness, 200 °C nozzle temperature, and 60 °C bed temperature were then chosen to print blocks. By changing these factors, finding the optimum build orientation in machining process would be hard. Each part needed at least two days for printing due to the long process. Therefore, each part was printed separately to reach the optimal conditions, such as reasonable cost and building time.

**Figure 5:**
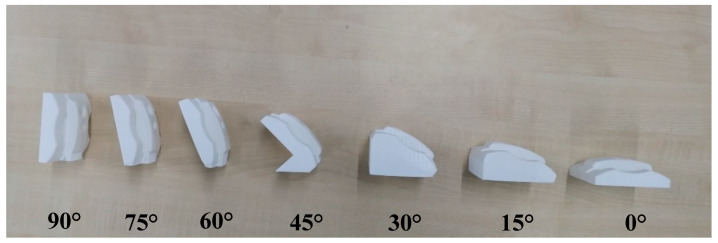
Printed complex Samples.

Meanwhile, a comparison between machined parts and complex printed parts was made by printing seven complex samples, which were identical to the bulk specimens’ orientation (see Figure 5). Due to the main goal of surface roughness, 20% infill was chosen for the complex samples as it would not have a significant effect on surface roughness [80,81]. Other parameters such as nozzle diameter, layer thickness, bed temperature, and build orientation were kept similar to the blocks.

**Figure 6:**
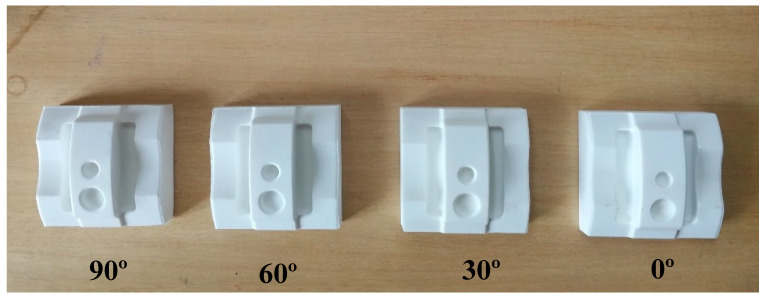
Machined samples.

**Figure 7:**
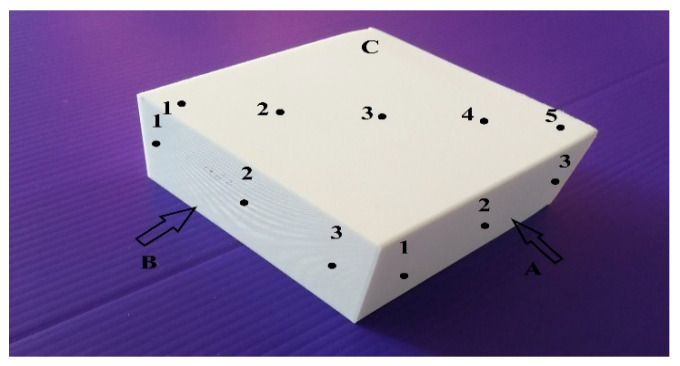
Blocks’ sides for surface measurement.

A DMU 70 Heidenhain iTNC 530 machine (DMG Mori, Bielefeld, Germany) was used to subtract any excess materials from the blocks in order to attain a complex shape. Four samples with 0°, 30°, 60°, and 90° build angles, respectively, were chosen for the machining process (see Figure 6) and five steps were used to machine the samples and reach the desired smoothness (see Table 2). Solid carbide cutters were used in this study, whereas the Powermill^®^ software (Autodesk, San Rafael, California, United States) was used to get G-codes. As mentioned, PLA absorbs water and moisture in the air. To eliminate this issue, pressurized air was used to make the samples cool during machining process. Side C (Figure 7) was chosen for machining process because of our design. Unfortunately, there have not been researches on smooth machining on PLA printed samples. Thus, a catalogue supplier was used for finishing steps and other parameters were like end-mill steps.

To determine the surface behaviour, measuring surface roughness parameters such as average roughness (*Ra*) and average peak to valley height (*Rz*) was necessary. Undoubtedly, roughness, waviness, and profile curve were the main factors affecting the final quality of the product texture. These parameters were evaluated by Mahr Perthometer S2 machine (Mahr Inc, Göttingen, Germany), whereby the printing samples showed different features in bulk parts. Therefore, each side of the parts yielded its features due to the layer-by-layer structure. Then, three zones of all rectangular parts were measured by the machine to investigate the *Ra* and *Rz* outcomes. The machine could measure the surface point by points, so five perpendicular points were chosen for zone C and three points were determined for zones A and B, respectively (see Figure 7).

**Figure 8:**
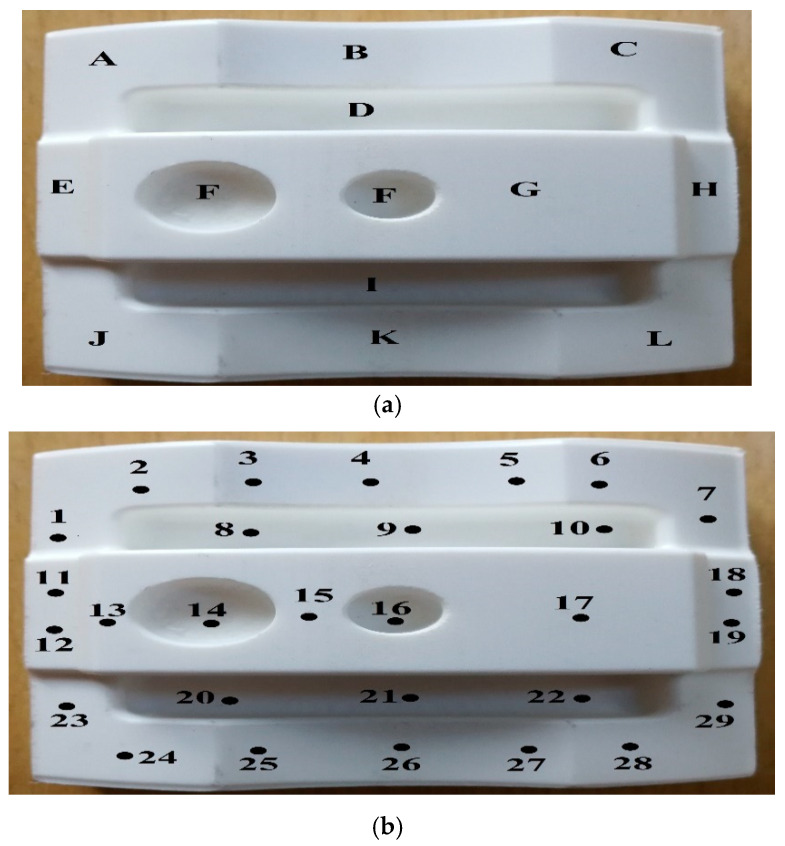
(**a**) Profiles for surface measurement; (**b**) Points for surface measurement.

**Figure 9:**
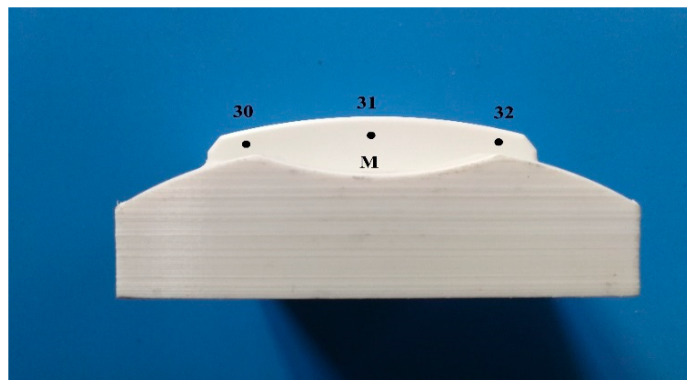
M side and points for surface measurement.

On the other hand, various points were chosen for complex parts to measure the surface roughness. Due to the non-constant geometry and various angles, each point showed specific feature and the surface angles had an extraordinary effect on surface smoothness [61]. Accordingly, for a reasonable and comprehensive comparison between printed and machined parts, the distributed points were chosen (see Figure 8 and Figure 9). Finally, a comparison between each profile to identify the average roughness and high surface quality among samples was done. Due to machine limitations in measuring the curves at 5.6 mm tracing length (*Lt*), the lowest *Lt* of 0.560 mm was chosen. The *Ra* and *Rz* values are affected by the decreasing *Lt* and the specific values are provided in Table 3 [82]. Based on Table 3, choosing a low Lt allows the *Ra* and *Rz* to play their roles in a lower frequency. In brief, each point was measured twice to find out *Ra* and *Rz* values. Due to the numerous values (e.g., 64 *Ra* and *Rz*) that were found for each sample, the average value was calculated for profiles. For example, in profile A, 8 *Ra* and *Rz* values were obtained while the average of 8 values were calculated for further examination. Then, each profile was analysed separately to find out its features and values. Finally, to find out how surface roughness changed in samples, the average value of all profiles was calculated.

## 4. Results and Discussion

**Figure 10:**
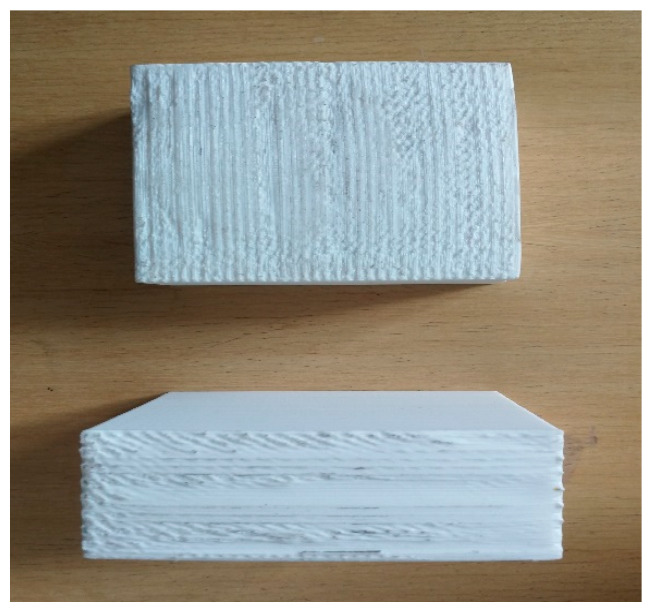
Staircase defect.

**Figure 11:**
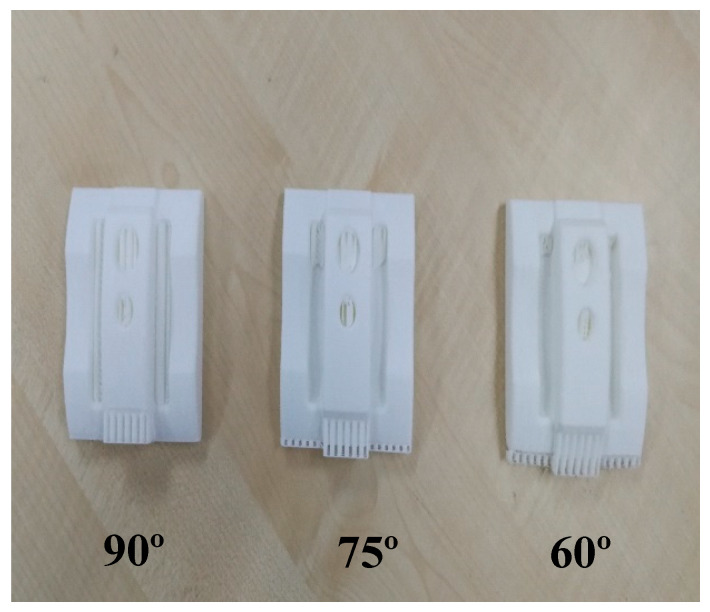
Supports in 60°, 75° and 90° samples.

The defects in machining and surface reaction during the printing process for blocks and complex samples were investigated in this study. Besides, the image of deformations and failures for both processes were recorded and discussed. Many parameters have an effect on surface roughness, such as build orientation, layer thickness, nozzle, and bed temperature. Meanwhile, some factors may affect surface quality during the printing process, such as product design, geometry, and material properties. For example, acrylonitrile butadiene styrene (ABS) has better surface characteristics compared to PLA materials [83]. Subsequently, stair-stepping effect and warpage are the main problems in FDM [84]. In this case, staircase defects were observed at the sides where the machine used supports in the blocks and complex samples during the printing process (see Figure 10). Therefore, post-processing techniques would be needed to make these sides smooth, such as sandpaper. On the other hand, for the 60°, 75°, and 90° orientations, the complex samples machine used supports inside the holes and slots, which would affect the surface roughness and prevent material drops during the printing process when building the specimens (see Figure 11).

**Figure 12:**
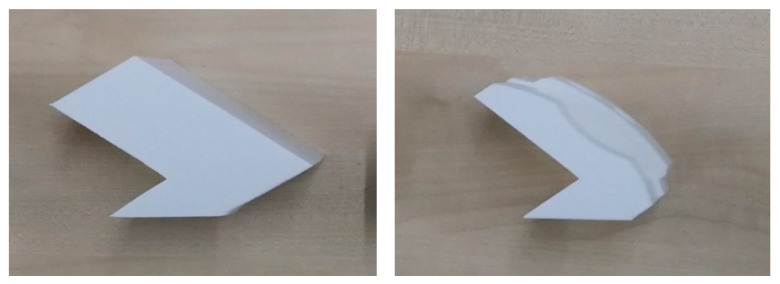
Support in 45° specimens.

As shown in Figure 12, due to the machine’s limitation to print bulk and complex parts in the 45° orientation, support was added to the specimens at the designing stage as the FDM machine could not build support itself as it had a sharp edge. Eventually, the support was removed by a grinder to make the bottom side flat. In brief, both processes were capable of producing small features, such as holes, fillets, and sluts.

### 4.1. Surface Profile Measurement

The tracing length was decreased to 0.560 mm in order to allow the machine to measure curves. Table 4 shows the average surface roughness (*Ra*) and average peak to valley height (*Rz*) of each block. Due to the angles between each layer in section A, *Ra* showed an opposing direction compared to the build orientation. In other words, by increasing the orientation, the value of *Ra* decreased step by step, which was why the 90° specimen had the lowest *Ra* and *Rz* (e.g., 0.648 and 3.13 µm) among other samples. In section B, both *Ra* and *Rz* decreased gradually from 0° to 60° before a sharp increase was seen for 75° and 90°, respectively. Meanwhile, in the C side, *Ra* increased slightly following an increasing build orientation. However, a slight reduction after 60° was seen in the stable orientation, namely 90°. Accordingly, the 0° sample had the lowest *Ra* while the 60° sample showed the highest *Ra*. Besides, the value of *Rz* became bigger in each orientation for side C. To conclude, each side showed specific behaviours in different orientations due to the layer binding and layer angles. However, after 60°, the specimens presented different values for *Ra* and *Rz*. This may occur due to different printing procedures as the top side in 0°, 15°, 30°, and 45° will become side surfaces in 60°, 75°, and 90°, and thus the features will change. As shown in Table 4, side C, which was the important side in printing process, the values of *Ra* and *Rz* were 0.690 and 3.294 µm which were the lowest value among printed blocks. Lowest *Ra* and *Rz* value, in this case, showed that sample had a smooth surface. It should be noted, the values are fluctuated in a small range between 0.6 to 2.5 µm for *Ra* and 3 to 10 µm for *Rz*. To continue this research, side C was chosen due to its machining design.

**Figure 13:**
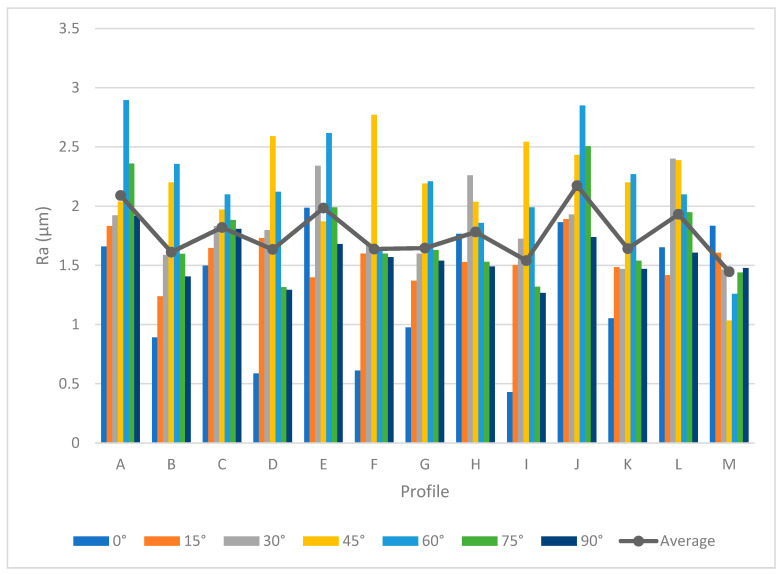
*Ra* comparison in complex printed samples.

**Figure 14:**
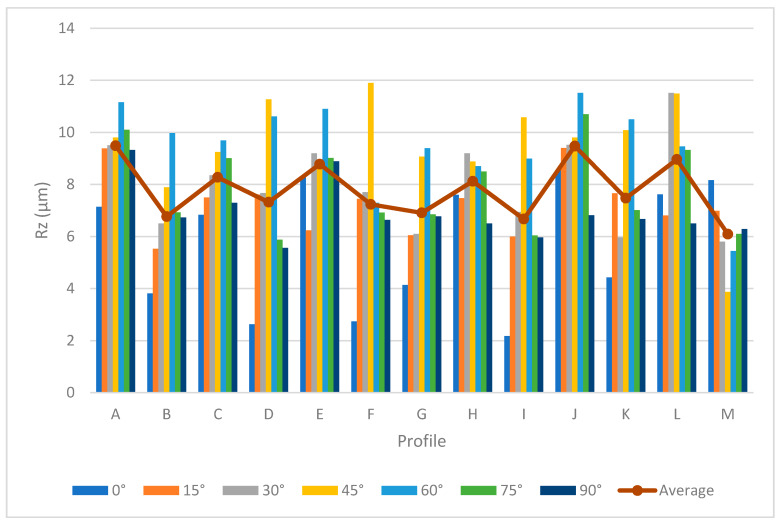
*Rz* comparison in complex printed samples.

Table 5 provides the *Ra* and *Rz* for different orientations. As mentioned before, the surface angle plays an important role in surface measurement, whereby each profile represents the specific values of *Ra* and *Rz*. To generate a comprehensive comparison between each profile, Figure 13 and Figure 14 illustrate the differences between each profile. Note that it was not possible to compare each sample due to different features. Therefore, the profiles in the samples were investigated separately.

**Figure 15:**
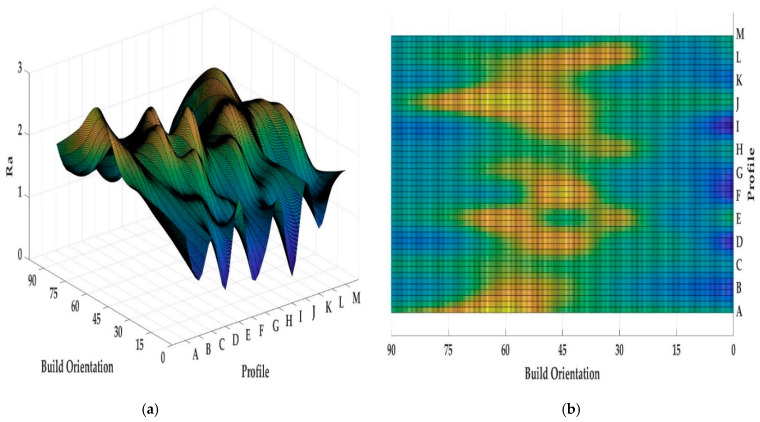
*(***a**) *Ra* values for printed complex samples in 3D graph, (**b**) *Ra* values for printed complex samples in 2D graph.

**Figure 16:**
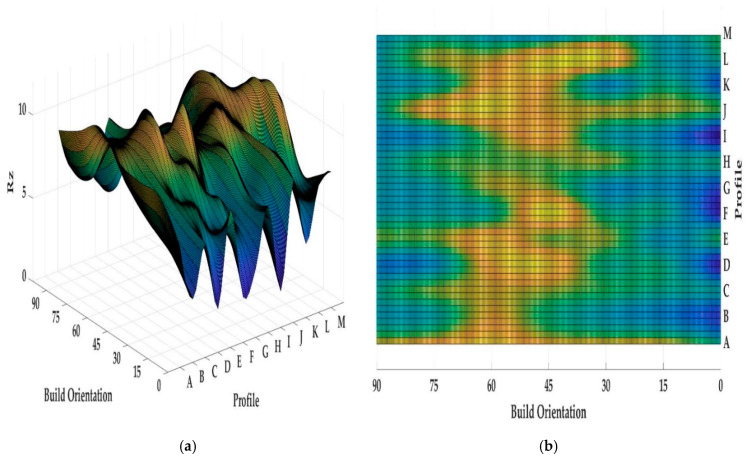
*(***a**) *Rz* values for printed complex samples in 3D graph, (**b**) *Rz* values for printed complex samples in 2D graph.

Both Figure 13 and Figure 14 show the values of *Ra* and *Rz* for each profile. First, the influence of infill density on surface roughness is not significant [80,81]. Furthermore, the 0° orientation had the lowest *Ra* and *Rz* roughness among all specimens, except in the E, H, J, L, and M profiles. However, on average, 0° orientation had the lowest *Ra* and *Rz* values while the variance of this sample was highest. Figure 15 and Figure 16 were conducted by MATLAB^®^ to make the differences clear. As shown in Figure 15 and Figure 16, the values of *Ra* and *Rz* were high for perpendicular samples while Figures show profiles had better quality in horizontal and vertical ones [48]. Based on information in Table 5, standard deviation for *Ra* and *Rz* was higher for 0° part among samples. It means each profile had different quality in this sample. On the other hand, the 60° orientation had the worst quality in the A, B, C, E, G, J, and K profiles similar to the block part. Holes and pockets, which consisted in F, D, and I profiles, had the worst surface roughness in 45°, while in 0°, the values for these profiles were the lowest. In E and H profiles, the values were different due to the slippery surfaces, whereas their *Ra* and *Rz* in the 15° and 90° samples were the lowest. In contrast, these values were the highest for the 60° and 30° samples. Therefore, the data showed that surface angle was affected in the printing procedure due to specific orientation and layer binding. In section L, the 15° and 30° samples had the worst surface smoothness, while they had the lowest outcomes in 90°. Surprisingly, in profile M, the 0° orientation had the worst *Ra* and *Rz* values 1.834 and 8.16 µm, while the graph showed that the 45° sample had the best surface roughness for this profile. All in all, by increasing the orientation, the *Ra* and *Rz* values were increased as well. Similar to the blocks, the surface roughness values decreased after 60°. Additionally, 0° had better surface quality compared to other specimens. Based on information in Table 5, the average values of *Ra* and *Rz* for profiles in samples are lowest for 0° part compared to other specimens which are 1.293 and 5.70 µm, respectively. It means, 0° part had the smoothest surface among others. Moreover, the samples illustrated that the FDM machine was capable to print details such as slopes, fillets, and small holes. In contrast, defects such as stair-step effect were seen in the printed parts, which were a major drawback in AM processes [19]. This result indicated that horizontal and vertical samples yielded better surface quality compared to perpendicular ones. In brief, build orientation had a great effect on sample in FDM process. Thus, by increasing the build orientation (e.g., 0° to 60°) the surface roughness became worse and, after 60° parts, surface roughness showed the opposite direction.

Both Figure 15 and Figure 16 show that, between 30° and 75° samples, the values of each profile were high compared to the 0°, 15°, and 90°. These differences between these specimens are clearer in Figure 16 because the values of *Rz* were high. Meanwhile, some profiles in these three samples were high due to the layer binding in the FDM process which is one the big challenges in AM [40].

**Figure 17:**
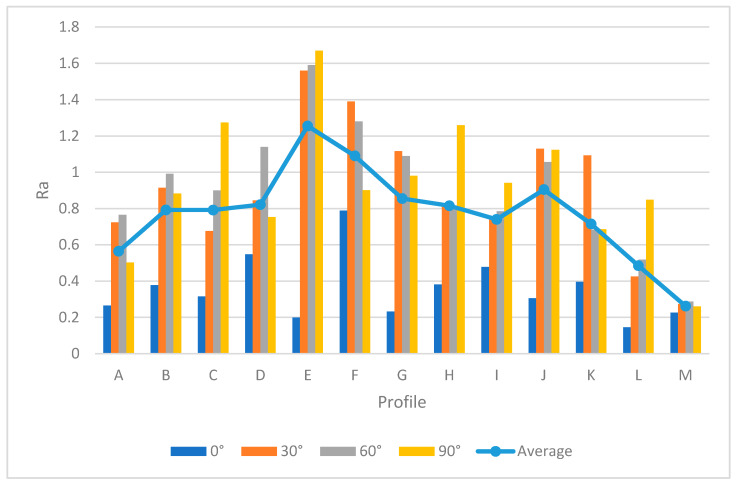
*Ra* comparison in machined samples.

**Figure 18:**
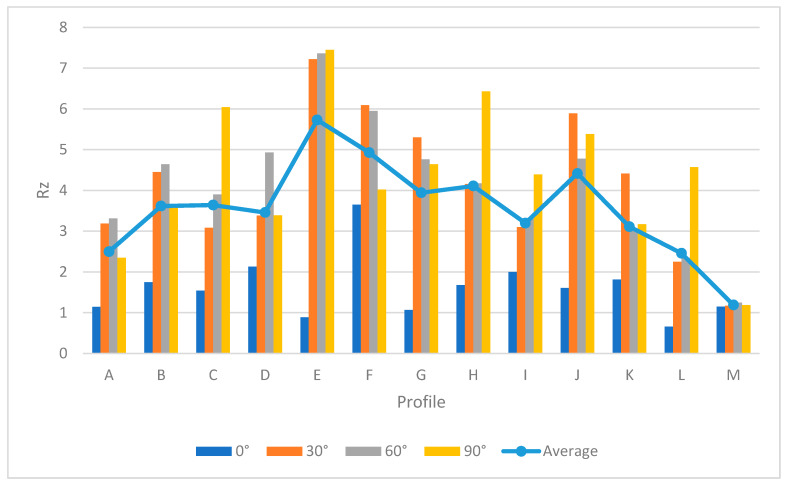
*Rz* comparison in machined samples.

The next step was to measure the surface roughness of machined samples in order to identify which had the lowest and highest values, respectively. To investigate the details of each profile, Table 6 shows the specific features of the samples. The *Ra* and *Rz* values were obtained by surface roughness machine and the tracing length was set at 0.560 mm to measure each point and identify the average value of each profile. Moreover, Figure 17 and Figure 18 show the characteristics of each profile accordingly.

**Figure 19:**
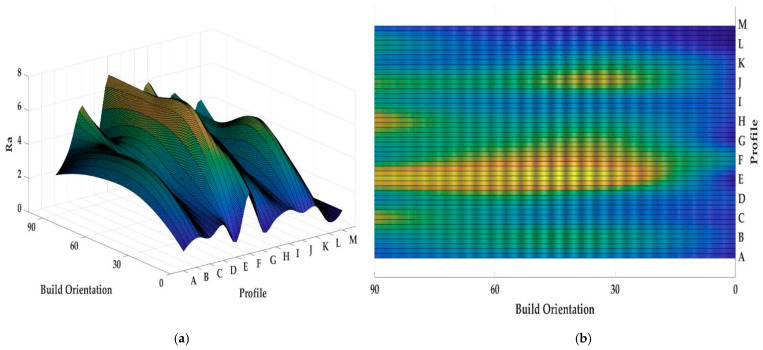
(**a**) *Ra* values for machined samples in 3D graph, (**b**) *Ra* values for machined samples in 2D graph.

**Figure 20:**
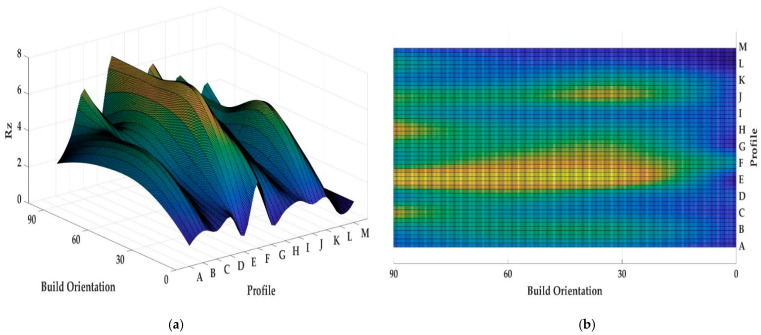
(**a**) *Rz* values for machined samples in 3D graph, (**b**) *Rz* values for machined samples in 2D graph.

**Figure 21:**
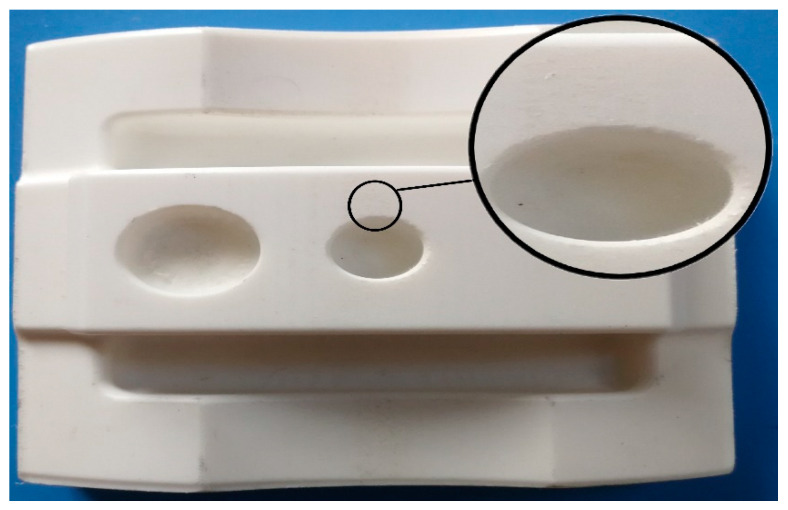
Defect in machining samples.

**Figure 22:**
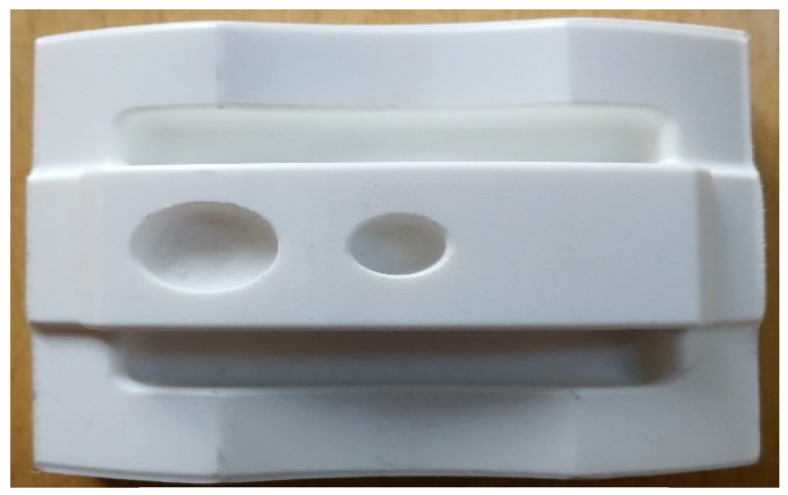
Surface texture in machined sample.

In Figure 17 and Figure 18, each profile showed different features in the machining process due to the layer-by-layer structure. Similar to the printed samples, 0° orientation showed better surface quality among the specimens due to its stable orientation. Same to the block and printed samples, 0° specimen had the lowest average values in profiles for *Ra* and *Rz* which were 0.358 µm and 1.622 µm, as shown in Table 6. Also, 0° sample had the lowest standard deviation (e.g., 0.17) and variance 0.03 for *Ra* value which shows the machining had a great impact on this sample. Furthermore, as the build orientation was increased, the values of *Ra* and *Rz* were increased as well. Slippery surfaces (i.e. E and H) had the highest values in the 90° sample, namely 1.67 µm and 1.260 µm (i.e., *Ra*) and 7.45 µm and 6.43 µm for *Rz*, respectively. In contrast, the holes had the worst surface quality in 30° (see Figure 19 and Figure 20). In profile M, the values of *Ra* and *Rz* were different compared to the printed samples, whereby the 0° sample had the best surface smoothness compared to other profiles. This might happen due to the side milling process, indicating that this process would lead to better surface quality compared to surface milling. As shown in Figure 19 and Figure 20 the highest value was for holes and it started to increase from 30° sample to 90°. Also, it can be seen from Figure 19 and Figure 20 that profile M had the best quality among other profiles. Meanwhile, one of the biggest limitations in plastics machining is material tendency to melt due to the high temperatures. Therefore, a pressurized air was used to reduce the temperature during the machining process. Astonishingly, each slut showed different values due to the machining defects, which would be discussed further. In Figure 21, some defects are seen in all machined samples and affected the final surface quality. The defect was similar to very small shattered materials or chips stuck to some parts of the surface and might occur due to poor feed rate or cutting speed in the final machining stage. As an example, this defect affected profile K in 30°. Therefore, the values of *Ra* and *Rz* were highest for profile K based on the Figure 17 and Figure 18. After the machining process, no layer structure was seen in the specimens (see Figure 22). Further, the surfaces were examined and showed better quality compared to the printing process in which elements such as fillets, sluts, slopes, and pockets showed a reasonable quality. Therefore, plastic machining could be done on small features for PLA material. Nevertheless, a comparison between the machined and printed samples was done to identify the differences and improvements between the two processes.

### 4.2. Comparison between Printed and Machined Samples

**Figure 23:**
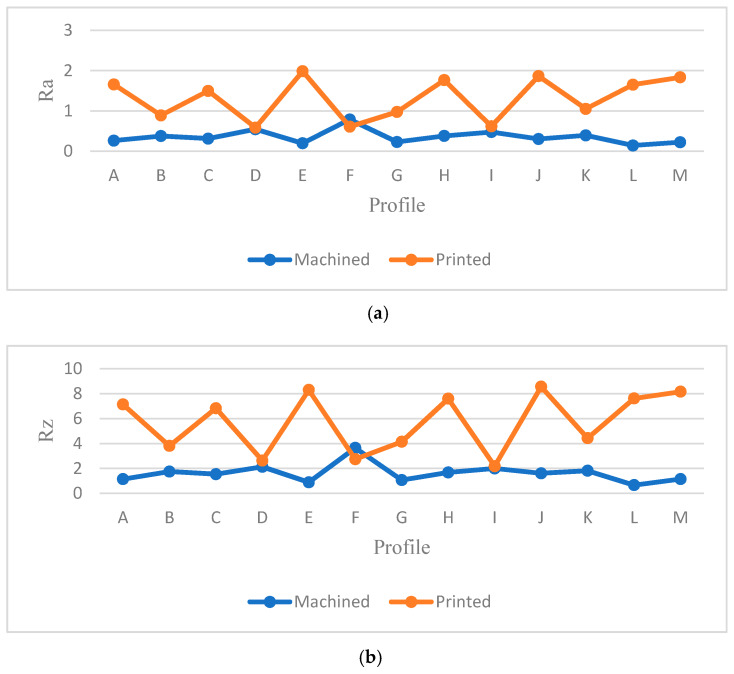
(**a**) *Ra* comparison between machined and printed for 0° specimens, (**b**) *Rz* comparison between machined and printed for 0° specimens.

**Figure 24:**
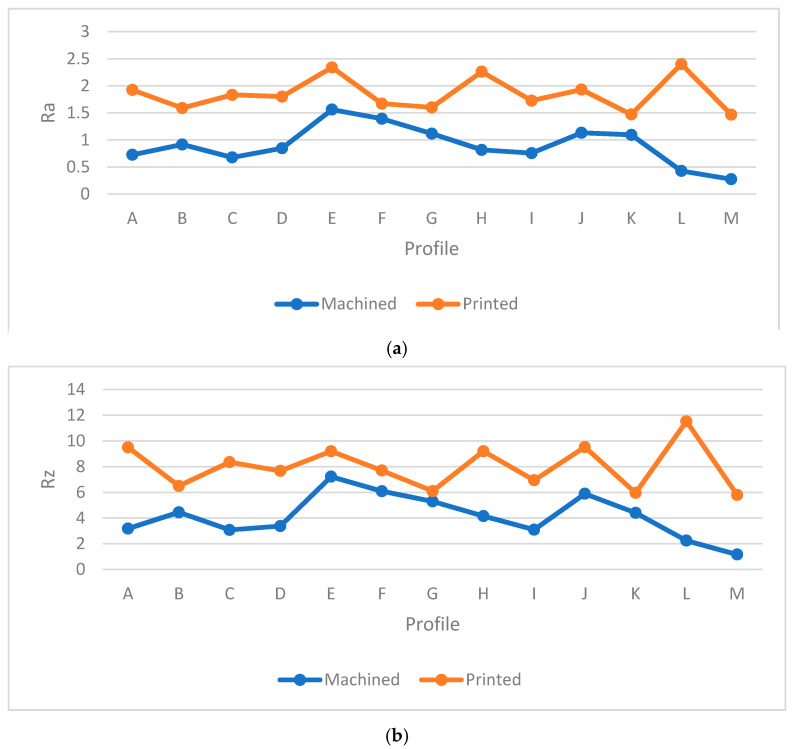
(**a**) *Ra* comparison between machined and printed for 30° specimens, (**b**) *Rz* comparison between machined and printed for 30° specimens.

**Figure 25:**
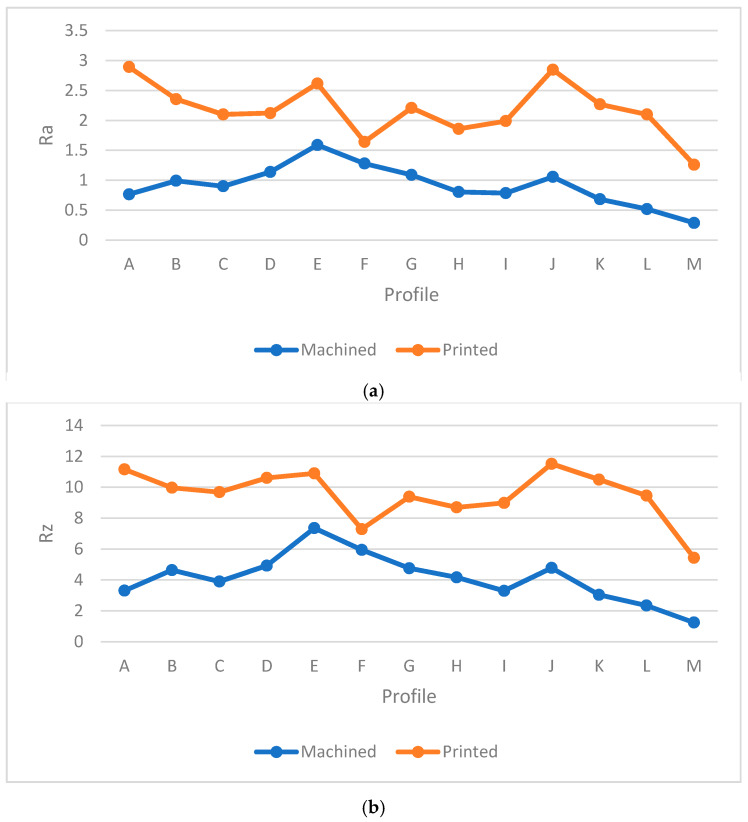
(**a**) *Ra* comparison between machined and printed for 60° specimens, (**b**) *Rz* comparison between machined and printed for 60° specimens.

**Figure 26:**
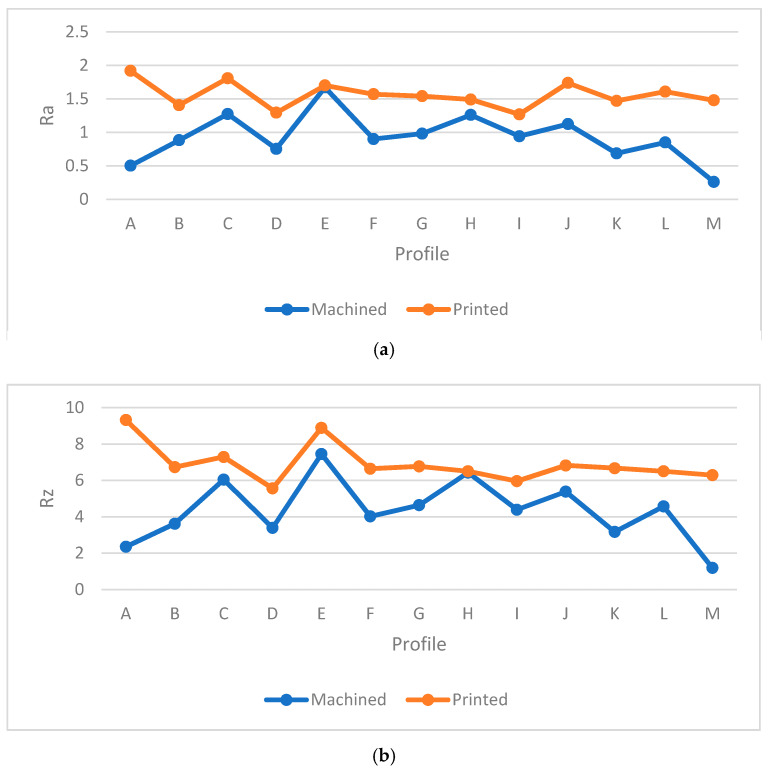
*(***a**) *Ra* comparison between machined and printed for 90° specimens, (**b**) *Rz* comparison between machined and printed for 90° specimens.

To investigate the differences between each sample, Figure 23, Figure 24, Figure 25 and Figure 26 are provided to indicate the surface roughness values of each profile and show the improvements of each sample in the machining procedure. As shown in Figure 23, for 0° sample, an improvement in the major profiles was seen, except for the holes and pocket, namely the F and I profiles. Moreover, the differences in values were insignificant in profile D. To investigate the extent to which these profiles are enhanced in the machining process compared to printing, the percentage of error is calculated for *Ra* and *Rz* as follows:

(1) $$Error\;\left(\%\right)=\frac{Printed\;value-Machined\;value\;}{Printed\;value}\times 100$$

Differences for all samples are provided in Table 7. First, the *Ra* values in the machined part fluctuated between 0.1 µm and 0.8 µm, which were very low compared to the printed samples. In profile F, the differences of *Ra* value between printed and machined samples were −29.13%. The values in the F section for the machining process were a little high due to the machining defects mentioned. In contrast, the results showed that flat surfaces yielded good quality compared to curves in the printing process. Nevertheless, a significant enhancement was seen in all profiles in the milling machining process. Further, it could be concluded that the FDM process was capable of printing flat surfaces of high quality in 0° due to the low values in the F, D, and I profiles. Moreover, profile E had the highest differences for *Ra* and *Rz*, namely 89.97% and 89.27%, respectively, compared to other sections. Therefore, a major improvement was seen in profile E. In machining, the holes had high *Ra* values, while profile E was the worst outcome in the printing sample. It should be noted that the surface angle could be affected in the printing process.

To verify this research, other samples were also investigated to identify the manner in which CNC machining affected the surface quality. The differences between all sections were positive and revealed how the machining process changed the roughness of the 30° sample. Besides, a major improvement was seen for profile L, yielding 82.25% of *Ra* value in 30°. Moreover, Figure 24 indicates the value of *Ra* and *Rz* for the 30° orientation sample. Each profile data showed that the machined part had better surface quality compared to the printed sample. Subsequently, in the machined sample, the pockets and holes had better surface quality; thus, the main problem in the 0° specimen was the defect of machining, which affected the surface quality and made the profiles rougher. In fact, by increasing the surface angle, the roughness increased as well. However, the sample’s texture was quite impressive, and each profile showed that machining had a great influence on thermoplastics (i.e., PLA).

Then, the 60° sample also showed an enhancement throughout the specimen similar to the 0° and 30° samples (see Figure 25). In this case, the holes yielded better surface roughness but section M had the best surface quality. Meanwhile, profile E in machining had the worst surface quality due to the 30° angle, while its holes revealed acceptable quality in the printed sample. However, profile M was the best compared to other profiles in both processes. Table 7 shows that it has the highest difference among other profiles in 60°, namely 77.22% and 77.02% for the *Ra* and *Rz*, respectively.

**Figure 27:**
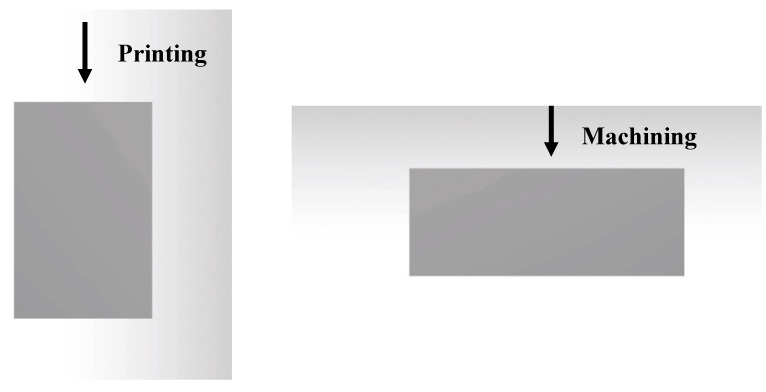
Printing and machining process for 90° orientation.

The last sample of 90° was investigated accordingly. Figure 26 shows that the value of *Ra* in the printed sample for profile E is close to the machined sample, with a surprising difference of 0.6%. However, other profiles yielded better surface smoothness, whereby a major variance was recorded for profile M. This profiled yielded values of 82.40% and 81.08% for its *Ra* and *Rz*, respectively, which were due to the low quality of the printed sample. Moreover, the different printing and machining processes as shown in Figure 27 result in most profiles having the worst surface quality in the perpendicular and vertical samples. Here, the printing started from one side while the machining removes the material from another side. Same to the 90° orientation, the 30° sample used a different machining angle and might affect the surface roughness.

In this section, all samples were examined to identify the manner in which the machining and printing processes affected surface roughness across different build orientations. It was found that HCM was a process capable of machining PLA blocks with high quality and accuracy. In the printing process, surface roughness was increased by increasing the build orientation but the roughness decreased step by step as upon reaching 90°, which was a stable orientation. The quality also became better compared to the perpendicular samples. Moreover, the results indicated 0° as the best orientation in blocks, complex printed samples, and complex machined specimens. By comparing the machined samples, no sign of layer-by-layer structure was found and each profile showed different characteristics due to the surface angle and curves. Additionally, this case showed profile M having the best quality of machined specimens due to side milling. Even though the printed samples showed a rougher surface, the process was capable of printing more complex products, which would not be possible by machining.

## 5. Conclusions

The surface quality and material attributes in the components produced via AM are important. In this study, the FDM 3D printer produced blocks and complex samples with different surface roughness, while works detailing different factors and parameters affecting the surfaces produced by AM were included. Seven printed blocks and complex printed samples both were printed by the FDM machine, which were then measured and analysed in terms of the surface roughness. Moreover, four blocks of 0°, 30°, 60°, and 90° orientations were machined by a CNC machine. The roughness of the samples was analysed in different build orientations and allowed a comparison in order to evaluate and measure the roughness and surface quality of printed and machined samples.

1. The main aim of this study was to identify the effect of machining 3D printed samples in different build orientations, where it was found that orientation had a great impact on surface roughness.
2. In this case, the roughness was highest in perpendicular such as 60° sample, and then it started to decrease gradually.
3. Machining had an extraordinary effect on surface roughness in which its samples had better surface quality compared to printed ones.
4. The results showed 0° sample had the best surface quality between both processes. The average values of *Ra* and *Rz* in profile for 0° specimen were 0.0690 µm and 3.294 µm in printed blocks, 1.293 µm and 5.7 µm in printed complex samples, and 0.358 µm and 1.622 µm in machined samples, respectively.
5. Sample with 90° orientation showed a higher *Ra* value compared to other machined samples due to the different printing and machining orientation as discussed before.
6. Shattered materials were found on machined samples surface after finishing process. Surfaces were affected due to the defects. A poor feed rate or cutting speed may cause this issue. It is recommended to find out the best machining parameters in the finishing process to eliminate this issue for further researches.
7. Holes in machining samples had the worst surface quality in this case due to the defect that is mentioned.
8. In machining process, profile M had the best surface quality. It means the machining parameters were suitable for side milling which lead to better surface.
9. Besides, this study showed that the surface roughness was better at certain angles than others, implying that one could improve the surface quality of the parts intended for 3D printing.
10. It should be noticed, the flat surfaces had better surface quality compared to slippery profiles.
11. By changing the build orientation, the slope surfaces are changed as well. This means that if the angle and orientation are aligned, the surface quality becomes better.
12. This study could be used as a guideline for the users of 3D printers requiring sample machining as a second process for better surface quality in different applications such as automotive, medical, and consumer products manufacturing which need good functional and tolerances. As a secondary process, CNC machining provides additional dimensional accuracy with tighter tolerances on additive parts while maintaining all the benefits of FDM. Popular applications, in this case, are jigs and fixtures which are made by FDM with light weight and ergonomically. Depending on the complexity of the jig or fixture, CNC machining is needed to line bore parts for alignment, face mill for smooth surfaces, or machine complementary metal parts. Another application is when products require complementary metal or plastic parts in different materials. This can be done by attaching machined plastic parts to the incomplete sample by pausing the FDM process to make an assembly product. Furthermore, hybrid manufacturing can be another application which is used FDM and CNC machining simultaneously.
13. Nevertheless, a lot of work is required and an expansive room for research is present in the field of FDM surface analysis. For example, no focus has been placed on layer thickness and its effects in the machining process. Therefore, future works can opt for samples that are 3D printed at different layer thicknesses to determine the effect on the surface roughness in the machining process. Moreover, different materials should be tested and analysed. The influence of different parameters in finishing machining and how they affect the surface can also be investigated in future studies.

## Figures and Tables

**Table 1:** Surface roughness parameters [51,52].

Parameter	Description	Equation
*Ra*	The arithmetical mean of the absolute values of *Z(x)* in a sampling length	$R_{a_{i}}=\frac{1}{l}\int_{0}^{l}\left|Zx\right|dx$
*Rq*	Root mean square average of the profile heights over the evaluation length	$R_{q_{i}}=\sqrt{\frac{1}{l}}\;\int_{0}^{l}Z^{2}\left(x\right)dx$
*Rsk*	Skewness uses the cube of the root mean square deviation to display the dimensionless cube of the sampling length *Z(x)*	$R_{sk}=\frac{1}{l\times R_{q_{i}}{}^{3}}\;\int_{0}^{l}Z^{3}\left(x\right)dx$
*Rku*	The peaks of the profile *(Zx)* about the mean line	$R_{sk}=\frac{1}{\;R_{q_{i}}{}^{4}}\;\int_{0}^{l}Z^{4}\left(x\right)dx$
*Rv*	The point along the sampling length at which the profile curve is lowest	$R_{v_{i}}=\begin{array}{c} Min\\ 1\leq j\leq m \end{array}Z_{v_{j}}$
*Rp*	The maximum value of peak height *Zp* is a sampling length on the profile curve	$R_{p_{i}}=\begin{array}{c} Max\\ 1\leq j\leq m \end{array}Z_{p_{j}}$
*Rz*	The absolute vertical distance between *Rp* and *Rv* along the sampling length	Rz=Rp+Rv

**Table 2:** Steps in machining process.

Machining Step	Process Type	Cutter (Ø) (mm)	Spindle Speed (rpm)	Cutting Speed (mm/min)	Feed Rate (mm/min)	Feed Per Tooth	Depth of Cut (mm)
1	End-mill	12	4377 [73]	165 [73]	1000 [73]	0.057	1
2	End-mill	5	3283	165	1000	0.057	1
3	Finishing top	5 Ball nose	10,504	165	1000	0.057	1
4	Finishing curves and pockets	5 Ball nose	10,504	165	1000	0.057	1
5	Finishing holes and whole model	6 Ball nose	10,504	165	1000	0.057	1

**Table 3:** Surface roughness elements [82].

Aperiodic Profiles		Cut-off Wavelength	Evaluation Length	Tracing Length
*Rz* (μm)	*Ra* (μm)	*λc* (mm)	*Ln* (mm)	*Lt* (mm)
<0.1	<0.02	0.08	0.40	0.56
0.1–0.5	0.02–0.1	0.25	1.25	1.75
0.5–10	0.1–2	0.8	4.0	5.6
10–50	2–10	2.5	12.5	17.5
>50	>10	8	40	56

**Table 4:** Blocks’ surface measurement.

Samples (BO)	Side	*Ra* (µm)	*Rz* (µm)	Samples (BO)	Side	*Ra* (µm)	*Rz*(µm)
0°	A	1.737	7.17	60°	A	1.541	5.89
	B	1.52	5.74		B	1.182	4.55
	C	0.690	3.294		C	2.149	9.602
15°	A	1.67	6.93	75°	A	1.36	4.86
	B	1.363	4.86		B	1.412	5.12
	C	1.059	3.824		C	1.825	9.421
30°	A	1.597	6.84	90°	A	0.648	3.13
	B	1.217	4.67		B	1.626	6.16
	C	1.386	4.848		C	1.687	8.036
45°	A	1.574	6.24	–	–	–	–
	B	1.192	4.63	–	–	–	–
	C	1.633	6.32	–	–	–	–

**Table 5:** Printed complex specimens’ surface measurement.

Sample (BO)	Profile	*Ra* (µm)	*Rz* (µm)	Sample (BO)	Profile	*Ra* (µm)	*Ra* (µm)
**0**°	A	1.660	7.14	**30**°	A	1.923	9.5
	B	0.891	3.81		B	1.589	6.50
	C	1.498	6.83		C	1.832	8.35
	D	0.587	2.63		D	1.799	7.67
	E	1.986	8.30		E	2.340	9.20
	F	0.611	2.74		F	1.670	7.70
	G	0.977	4.14		G	1.600	6.10
	H	1.766	7.6		H	2.26	9.2
	I	0.620	2.18		I	1.725	6.94
	J	1.865	8.56		J	1.93	9.52
	K	1.053	4.43		K	1.470	5.96
	L	1.653	7.62		L	2.40	11.52
	M	1.834	8.16		M	1.467	5.80
	**Average**	**1.293**	**5.70**		**Average**	**1.846**	**7.99**
	**Std dev.**	**0.55**	**2.41**		**Std dev.**	**0.32**	**1.74**
	**Variance**	**0.3**	**5.82**		**Variance**	**0.1**	**3.01**
**15**°	A	1.832	9.38	**45**°	A	2.039	9.8
	B	1.24	5.53		B	2.200	7.89
	C	1.647	7.50		C	1.971	9.25
	D	1.732	7.61		D	2.591	11.27
	E	1.398	6.24		E	1.873	8.90
	F	1.600	7.45		F	2.770	11.90
	G	1.371	6.05		G	2.190	9.07
	H	1.529	7.47		H	2.038	8.88
	I	1.504	6.00		I	2.543	10.58
	J	1.890	9.40		J	2.433	9.8
	K	1.486	7.66		K	2.2	10.08
	L	1.418	6.81		L	2.388	11.49
	M	1.607	6.99		M	1.034	3.87
	**Average**	**1.558**	**7.23**		**Average**	**2.174**	**9.44**
	**Std dev.**	**0.19**	**1.18**		**Std dev.**	**0.43**	**2.04**
	**Variance**	**0.03**	**1.40**		**Variance**	**0.19**	**4.15**
**60**°	A	2.894	11.16	**90**°	A	1.919	9.32
	B	2.356	9.97		B	1.406	6.73
	C	2.100	9.69		C	1.808	7.29
	D	2.122	10.61		D	1.293	5.56
	E	2.617	10.90		E	1.70	8.89
	F	1.642	7.29		F	1.57	6.64
	G	2.210	9.39		G	1.540	6.77
	H	1.859	8.7		H	1.490	6.50
	I	1.990	8.99		I	1.268	5.96
	J	2.849	11.52		J	1.738	6.82
	K	2.269	10.5		K	1.470	6.67
	L	2.1	9.46		L	1.608	6.50
	M	1.260	5.44		M	1.478	6.29
	**Average**	**2.175**	**9.50**		**Average**	**1.56**	**6.91**
	**Std dev.**	**0.45**	**1.67**		**Std dev.**	**0.19**	**1.06**
	**Variance**	**0.21**	**2.80**		**Variance**	**0.04**	**1.13**
**75**°	A	2.36	10.1				
	B	1.597	6.93				
	C	1.883	9.01				
	D	1.317	5.88				
	E	1.990	9.02				
	F	1.60	6.92				
	G	1.630	6.85				
	H	1.530	8.50				
	I	1.32	6.04				
	J	2.506	10.70				
	K	1.54	7.01				
	L	1.949	9.32				
	M	1.44	6.85				
	**Average**	**2.174**	**7.87**				
	**Std dev.**	**0.37**	**1.64**				
	**Variance**	**0.14**	**2.68**				

**Table 6:** Machined specimens’ surface measurement.

Sample (BO)	Profile	*R_a_* (µm)	*R_z_* (µm)	Sample (BO)	Profile	*R_a_* (µm)	*R_z_* (µm)
**0**°	A	0.265	1.145	**60**°	A	0.765	3.31
	B	0.378	1.75		B	0.992	4.64
	C	0.315	1.54		C	0.9	3.9
	D	0.548	2.13		D	1.139	4.93
	E	0.199	0.89		E	1.59	7.36
	F	0.789	3.65		F	1.28	5.95
	G	0.232	1.07		G	1.09	4.76
	H	0.382	1.68		H	0.804	4.17
	I	0.478	2		I	0.785	3.3
	J	0.306	1.61		J	1.056	4.78
	K	0.396	1.814		K	0.683	3.04
	L	0.145	0.66		L	0.519	2.35
	M	0.226	1.15		M	0.287	1.25
	**Average**	**0.358**	**1.622**		**Average**	**0.914**	**4.13**
	**Std dev.**	**0.17**	**0.75**		**Std dev.**	**0.34**	**1.57**
	**Variance**	**0.03**	**0.57**		**Variance**	**0.12**	**2.46**
**30**°	A	0.724	3.185	**90**°	A	0.502	2.35
	B	0.915	4.45		B	0.883	3.62
	C	0.676	3.08		C	1.274	6.04
	D	0.845	3.38		D	0.753	3.39
	E	1.56	7.22		E	1.67	7.45
	F	1.39	6.09		F	0.901	4.02
	G	1.116	5.3		G	0.981	4.64
	H	0.815	4.155		H	1.26	6.43
	I	0.755	3.1		I	0.942	4.39
	J	1.13	5.89		J	1.124	5.38
	K	1.093	4.41		K	0.686	3.17
	L	0.426	2.25		L	0.849	4.57
	M	0.274	1.17		M	0.26	1.19
	**Average**	**0.901**	**4.12**		**Average**	**0.929**	**4.35**
	**Std dev.**	**0.36**	**1.68**		**Std dev.**	**0.36**	**1.71**
	**Variance**	**0.14**	**2.83**		**Variance**	**0.12**	**2.91**

**Table 7:** *Ra* & *Rz* differences between machined and printed samples.

Sample	Profile	*R_a_* (%)	*R_z_* (%)	Sample	Profile	*R_a_* (%)	*R_z_* (%)
0°	A	84.04	83.96	30°	A	62.35	66.47
	B	57.57	54.06		B	42.41	31.53
	C	78.97	77.45		C	63.10	63.11
	D	6.64	19.01		D	53.02	55.93
	E	89.97	89.27		E	33.33	21.52
	F	−29.13	−33.21		F	16.76	20.90
	G	76.25	74.15		G	30.25	13.11
	H	78.36	77.89		H	63.93	54.83
	I	22.90	8.25		I	56.23	55.33
	J	83.59	81.19		J	41.45	38.13
	K	62.39	59.05		K	25.64	26.00
	L	91.22	91.33		L	82.25	80.46
	M	87.67	85.90		M	81.32	79.82
60°	A	73.56	70.34	90°	A	73.84	74.78
	B	57.89	53.46		B	37.19	46.21
	C	57.14	59.75		C	29.53	17.14
	D	46.32	53.53		D	41.76	39.02
	E	39.24	32.47		E	0.60	16.19
	F	22.04	18.38		F	42.61	39.45
	G	50.67	49.30		G	36.29	31.46
	H	56.75	52.06		H	15.43	1.07
	I	60.55	63.29		I	25.70	26.34
	J	62.93	58.50		J	35.32	21.11
	K	69.89	71.04		K	53.33	52.47
	L	75.28	75.15		L	47.20	29.69
	M	77.22	77.02		M	82.40	81.08

## References

[1] Bralla J.G. (1999). Design for Manufacturability Handbook.

[2] Kalpakjian S. (2009). Manufacturing Engineering and Technology.

[3] Ghaffar S.H., Corker J., Fan M. (2018). Additive manufacturing technology and its implementation in construction as an eco-innovative solution. Autom. Constr..

[4] Bose S., Ke D., Sahasrabudhe H., Bandyopadhyay A. (2018). Additive manufacturing of biomaterials. Prog. Mater. Sci..

[5] Goh G., Agarwala S., Dikshit V., Sing S., Yeong W.Y., Goh G. (2017). Additive manufacturing in unmanned aerial vehicles (UAVs): Challenges and potential. Aerosp. Sci. Technol..

[6] Cuellar J., Smit G., Plettenburg D., Zadpoor A. (2018). Additive manufacturing of non-assembly mechanisms. Addit. Manuf..

[7] Calignano F., Manfredi D., Ambrosio E.P., Biamino S., Lombardi M., Atzeni E., Salmi A., Minetola P., Iuliano L., Fino P. (2017). Overview on Additive Manufacturing Technologies. Proc. IEEE.

[8] Francois M., Sun A., King W., Henson N., Tourret D., Bronkhorst C., Carlson N., Newman C., Haut T., Bakosi J. (2017). Modeling of additive manufacturing processes for metals: Challenges and opportunities. Curr. Opin. Solid State Mater. Sci..

[9] Boparai K.S., Singh R., Singh H. (2016). Development of rapid tooling using fused deposition modeling: A review. Rapid Prototyp. J..

[10] Marwah O., Sharif S., Ibrahim M., Mohamad E., Idris M. (2016). Direct rapid prototyping evaluation on multijet and fused deposition modeling patterns for investment casting. Proc. Inst. Mech. Eng. Part L J. Mater. Des. Appl..

[11] Gebhardt A. (2016). Hötter, J.-S. 4—Rapid Prototyping. Additive Manufacturing.

[12] Pham D.T., Gault R. (1998). A comparison of rapid prototyping technologies. Int. J. Mach. Tools Manuf..

[13] Ariffin M.K.A.M., Sukindar N.A., Baharudin B.H.T., A Jaafar C.N., Ismail M.I.S. (2018). Slicer Method Comparison Using Open-source 3D Printer. Proceedings of the IOP Conference Series: Earth and Environmental Science.

[14] Ngo T., Kashani A., Imbalzano G., Nguyen Q.T., Hui D. (2018). Additive manufacturing (3D printing): A review of materials, methods, applications and challenges. Compos. Part B: Eng..

[15] Chia H.N., Wu B.M. (2015). Recent advances in 3D printing of biomaterials. J. Boil. Eng..

[16] Frazier W.E. (2014). Metal Additive Manufacturing: A Review. J. Mater. Eng. Perform..

[17] Gibson I., Rosen D.W., Stucker B. (2014). Additive Manufacturing Technologies.

[18] Wong K.V., Hernandez A. (2012). A Review of Additive Manufacturing. ISRN Mech. Eng..

[19] Panda B., Shankhwar K., Garg A., Jian Z. (2016). Performance evaluation of warping characteristic of fused deposition modelling process. Int. J. Adv. Manuf. Technol..

[20] Matsuzaki R., Ueda M., Namiki M., Jeong T.-K., Asahara H., Horiguchi K., Nakamura T., Todoroki A., Hirano Y. (2016). Three-dimensional printing of continuous-fiber composites by in-nozzle impregnation. Sci. Rep..

[21] Ning F., Cong W., Qiu J., Wei J., Wang S. (2015). Additive manufacturing of carbon fiber reinforced thermoplastic composites using fused deposition modeling. Compos. Part B Eng..

[22] Dul S., Fambri L., Pegoretti A. (2016). Fused deposition modelling with ABS–graphene nanocomposites. Compos. Part A Appl. Sci. Manuf..

[23] Panda B., Unluer C., Tan M.J. (2018). Investigation of the rheology and strength of geopolymer mixtures for extrusion-based 3D printing. Cem. Concr. Compos..

[24] Jabbari A., Abrinia K. (2018). Developing thixo-extrusion process for additive manufacturing of metals in semi-solid state. J. Manuf. Process.

[25] Hsieh P.C., Tsai C.H., Liu B.H., Wei W.C.J., Wang A.B., Luo R.C. (2016). 3D printing of low melting temperature alloys by fused deposition modeling. Proceedings of the 2016 IEEE International Conference on Industrial Technology (ICIT).

[26] Le Duigou A., Castro M., Bevan R., Martin N. (2016). 3D printing of wood fibre biocomposites: From mechanical to actuation functionality. Mater. Des..

[27] Kruth J.-P., Leu M., Nakagawa T. (1998). Progress in Additive Manufacturing and Rapid Prototyping. CIRP Ann..

[28] Kuo C.-C., Wu Y.-R., Li M.-H., Wu H.-W. (2018). Minimizing warpage of ABS prototypes built with low-cost fused deposition modeling machine using developed closed-chamber and optimal process parameters. Int. J. Adv. Manuf. Technol..

[29] Alsoufi M., El-Sayed A. (2017). Warping Deformation of Desktop 3D Printed Parts Manufactured by Open Source Fused Deposition Modeling (FDM) System. Int. J. Mech. Mechatron. Eng..

[30] Bourell D., Kruth J.P., Leu M., Levy G., Rosen D., Beese A.M., Clare A. (2017). Materials for additive manufacturing. CIRP Annals.

[31] Choi J.-W., Medina F., Kim C., Espalin D., Rodríguez D., Stucker B., Wicker R. (2011). Development of a mobile fused deposition modeling system with enhanced manufacturing flexibility. J. Mater. Process. Technol..

[32] Wulle F., Coupek D., Schäffner F., Verl A., Oberhofer F., Maier T. (2017). Workpiece and Machine Design in Additive Manufacturing for Multi-Axis Fused Deposition Modeling. Procedia CIRP.

[33] Isa M.A., Lazoglu I. (2019). Five-axis additive manufacturing of freeform models through buildup of transition layers. J. Manuf. Syst..

[34] Faludi J., Bayley C., Bhogal S., Iribarne M. (2015). Comparing environmental impacts of additive manufacturing vs traditional machining via life-cycle assessment. Rapid Prototyp. J..

[35] Yan Q., Dong H.-H., Su J., Han J., Song B., Wei Q., Shi Y. (2018). A Review of 3D Printing Technology for Medical Applications. Engineering.

[36] Domingo-Espin M., Puigoriol-Forcada J.M., García-Granada A.A., Llumà J., Borrós S., Reyes G. (2015). Mechanical property characterization and simulation of fused deposition modeling Polycarbonate parts. Mater. Des..

[37] Zhao J., Zhang M., Zhu Y., Li X., Wang L. (2018). A Novel Optimization Design Method of Additive Manufacturing Oriented Porous Structures. Proceedings of the ASME 2018 International Mechanical Engineering Congress and Exposition.

[38] McCullough E.J., Yadavalli V.K. (2013). Surface modification of fused deposition modeling ABS to enable rapid prototyping of biomedical microdevices. J. Mater. Process. Technol..

[39] Alauddin M., Choudhury I., El Baradie M., Hashmi M. (1995). Plastics and their machining: A review. J. Mater. Process. Technol..

[40] Salmi M., Ituarte I.F., Chekurov S., Huotilainen E. (2016). Effect of build orientation in 3D printing production for material extrusion, material jetting, binder jetting, sheet object lamination, vat photopolymerisation, and powder bed fusion. Int. J. of Collab. Enterp..

[41] Peng T., Xu X. (2014). Energy-efficient machining systems: A critical review. Int. J. Adv. Manuf. Technol..

[42] Song H.-C., Ray N., Sokolov D., Lefebvre S. (2017). Anti-aliasing for fused filament deposition. Comput. Des..

[43] Benardos P., Vosniakos G.-C. (2003). Predicting surface roughness in machining: A review. Int. J. Mach. Tools Manuf..

[44] Caggiano A. (2018). Machining of Fibre Reinforced Plastic Composite Materials. Materials.

[45] Hsu T.-K., Zeren E. (2004). Effects of cutting edge geometry, workpiece hardness, feed rate and cutting speed on surface roughness and forces in finish turning of hardened AISI H13 steel. Int. J. Adv. Manuf. Technol..

[46] Bhushan B. (2000). Surface Roughness Analysis and Measurement Techniques. Modern Tribology Handbook, Two Volume Set.

[47] Black J.T., Kohser R.A., DeGarmo E.P. (2017). DeGarmo’s Materials and Processes in Manufacturing.

[48] Taufik M., Jain P.K. (2016). A Study of Build Edge Profile for Prediction of Surface Roughness in Fused Deposition Modeling. J. Manuf. Sci. Eng..

[49] Henerichs M., Voß R., Küster F., Wegener K. (2015). Machining of carbon fiber reinforced plastics: Influence of tool geometry and fiber orientation on the machining forces. CIRP J. Manuf. Sci. Technol..

[50] Taufik M., Jain P.K. (2017). Laser assisted finishing process for improved surface finish of fused deposition modelled parts. J. Manuf. Process.

[51] Leach R. (2013). Characterisation of Areal Surface Texture.

[52] Sauri J., Suñé-Negre J., Diaz J., Vilana J., Millan D., Ticó J., Miñarro M., Pérez-Lozano P., García-Montoya E. (2015). Relationships between surface free energy, surface texture parameters and controlled drug release in hydrophilic matrices. Int. J. Pharm..

[53] Ali F., Chowdary B.V., Maharaj J. (2014). Influence of some process parameters on build time, material consumption, and surface roughness of FDM processed parts: Inferences based on the Taguchi design of experiments. Proceedings of the 2014 IACJ/ISAM Joint International Conference.

[54] Dey A., Yodo N. (2019). A Systematic Survey of FDM Process Parameter Optimization and Their Influence on Part Characteristics. J. Manuf. Mater. Process..

[55] Khan M.S., Dash J.P. (2018). Enhancing Surface Finish of Fused Deposition Modelling Parts. 3D Printing and Additive Manufacturing Technologies.

[56] Chen Y.-F., Wang Y.-H., Tsai J.-C. (2019). Enhancement of surface reflectivity of fused deposition modeling parts by post-processing. Opt. Commun..

[57] Adel M., Abdelaal O., Gad A., Nasr A.B., Khalil A. (2018). Polishing of fused deposition modeling products by hot air jet: Evaluation of surface roughness. J. Mater. Process. Technol..

[58] Thrimurthulu K., Pandey P.M., Reddy N.V. (2004). Optimum part deposition orientation in fused deposition modeling. Int. J. Mach. Tools Manuf..

[59] Bellini A., Güçeri S. (2003). Mechanical characterization of parts fabricated using fused deposition modeling. Rapid Prototyp. J..

[60] Boschetto A., Bottini L. (2014). Accuracy prediction in fused deposition modeling. Int. J. Adv. Manuf. Technol..

[61] Ahn D., Kweon J.-H., Kwon S.-M., Song J., Lee S. (2009). Representation of surface roughness in fused deposition modeling. J. Mater. Process. Technol..

[62] Durgun I., Ertan R. (2014). Experimental investigation of FDM process for improvement of mechanical properties and production cost. Rapid Prototyp. J..

[63] Zhang Y., Chou K. (2008). A parametric study of part distortions in fused deposition modelling using three-dimensional finite element analysis. Proc. Inst. Mech. Eng. Part B J. Eng. Manuf..

[64] Salonitis K., D’Alvise L., Schoinochoritis B., Chantzis D. (2015). Additive manufacturing and post-processing simulation: Laser cladding followed by high speed machining. Int. J. Adv. Manuf. Technol..

[65] Hällgren S., Pejryd L., Ekengren J. (2016). Additive Manufacturing and High Speed Machining -cost Comparison of short Lead Time Manufacturing Methods. Procedia CIRP.

[66] Kumbhar N.N., Mulay A.V. (2016). Post Processing Methods used to Improve Surface Finish of Products which are Manufactured by Additive Manufacturing Technologies: A Review. J. Inst. Eng. India Ser. C.

[67] Pandey P.M., Reddy N.V., Dhande S.G. (2003). Improvement of surface finish by staircase machining in fused deposition modeling. J. Mater. Process. Technol..

[68] Boschetto A., Bottini L., Veniali F. (2016). Finishing of Fused Deposition Modeling parts by CNC machining. Robot. Comput. Manuf..

[69] Chohan J.S., Singh R. (2017). Pre and post processing techniques to improve surface characteristics of FDM parts: A state of art review and future applications. Rapid Prototyp. J..

[70] Shukor J.A., Said S., Harun R., Husin S., Kadir A. (2016). Optimising of machining parameters of plastic material using Taguchi method. Adv. Mater. Process. Technol..

[71] Dhokia V., Kumar S., Vichare P., Newman S., Allen R.D. (2008). Surface roughness prediction model for CNC machining of polypropylene. Proc. Inst. Mech. Eng. Part B J. Eng. Manuf..

[72] Raju K.V.M.K., Janardhana G.R., Kumar P.N., Rao V.D.P. (2011). Optimization of cutting conditions for surface roughness in CNC end milling. Int. J. Precis. Eng. Manuf..

[73] Pămărac R.G., Petruse R.E. (2018). Study Regarding the Optimal Milling Parameters for Finishing 3D Printed Parts from ABS and PLA Materials. Acta Univ. Cibiniensis.

[74] Prakasvudhisarn C., Kunnapapdeelert S., Yenradee P. (2008). Optimal cutting condition determination for desired surface roughness in end milling. Int. J. Adv. Manuf. Technol..

[75] Taufik M., Jain P.K. (2016). CNC-assisted selective melting for improved surface finish of FDM parts. Taylor Fr. J. Virtual Phys. Prototyp..

[76] Krolczyk G., Raos P., Legutko S. (2014). Experimental analysis of surface roughness and surface texture of machined and fused deposition modelled parts. Tehnicki Vjesnik.

[77] Pérez M., Medina-Sanchez G., Collado A.G., Gupta M.K., Carou D. (2018). Surface Quality Enhancement of Fused Deposition Modeling (FDM) Printed Samples Based on the Selection of Critical Printing Parameters. Materials.

[78] Taufik M., Jain P.K. (2013). Role of build orientation in layered manufacturing: A review. Int. J. Manuf. Technol. Manag..

[79] Pandey P.M., Thrimurthulu K. (2004). Optimal part deposition orientation in FDM by using a multicriteria genetic algorithm. Int. J. Prod. Res..

[80] Reddy V., Flys O., Chaparala A., Berrimi C.E., Amogh V., Rosen B.G. (2018). Study on surface texture of Fused Deposition Modeling. Procedia Manuf..

[81] Peng T., Yan F. (2018). Dual-objective Analysis for Desktop FDM Printers: Energy Consumption and Surface Roughness. Procedia CIRP.

[82] Quinten M. (2019). A Practical Guide to Surface Metrology. Psychological and Social Measurement.

[83] Hyndhavi D., Babu G.R., Murthy S.B. (2018). Investigation of Dimensional Accuracy and Material Performance in Fused Deposition Modeling. Mater. Today: Proc..

[84] Armillotta A., Bellotti M., Cavallaro M. (2018). Warpage of FDM parts: Experimental tests and analytic model. Robot. Comput. Manuf..

